# Exploring the Role of Thioredoxin system in Cancer Immunotherapy

**DOI:** 10.7150/jca.98306

**Published:** 2025-01-01

**Authors:** Lin Sun, Anni Yu, Yang Yang, Zhiruo Wang, Wenqian Wang, Lan Luo

**Affiliations:** 1 State Key Laboratory of Pharmaceutical Biotechnology, School of Life Sciences, Nanjing University, Nanjing, Jiangsu, China.; 2 Wenzhou Medical University, Wenzhou, China.; 3 Department of Plastic Surgery, The Second Affiliated Hospital and Yuying Children's Hospital of Wenzhou Medical University, Wenzhou, China.

**Keywords:** Trx system, Tumor immune, T cell dysfunction, Immunotherapy response, Clinical biomarker.

## Abstract

**Purpose:** The thioredoxin (Trx) system is integral to redox regulation and participates in several physiological processes, including tumor growth, immune response, and stem cell differentiation. We have performed a comprehensive and holistic analysis of the Trx system in tumor immunity in this study.

**Methods:** A study using the Human Protein Atlas (HPA) and Clinical Proteomic Tumor Analysis Consortium (CPTAC) databases was conducted to determine the expression and distribution of Trx system proteins. To explore and validate the correlation between Trx system expression levels and tumor progression, GTEx and TCGA datasets were used. Western blotting was used to measure Trx system expression in lung cancer cell lines, while MTT assays were used to measure cell proliferation. The Kaplan-Meier plotter database was used to explore the association between the Trx system and survival outcome of patients in pan-cancer. GO and KEGG enrichment analyses of the Trx system were performed. Next, we analyzed how the Trx system related to immune activation. Using TIDE and TISMO databases, we predicted immunotherapy responses.

**Results:** An abnormal expression of the Trx system is observed in cancer cells. Interference with the Trx system with siRNA or inhibitors significantly inhibits tumor cell growth, suggesting the Trx system is crucial to tumor growth. Through a broad cohort of different cancer types, we explored the prominent role of genes in the Trx system. The Trx system showed a relatively consistent aberrant expression in pan-cancer, correlated closely with clinical prognosis. Interestingly, the Trx system was highly correlated with the clinical prognosis in pan-cancer, as well as immunity and metabolism. The abnormal expression of the thioredoxin system was positively correlated with the expression of genes associated with immune infiltration and with a decrease in survival. The Trx system was also associated with immune response to immunotherapy.

**Conclusion:** The Trx system is a good predictor of both survival and the efficacy of immunotherapy, as well as clinical prognosis.

## Introduction

An in-depth analysis of tumor incidence and development provides valuable indicators and a solid foundation for targeted therapy, as well as spurs progress in targeted therapy. In order to adapt to targeted therapeutic approaches, tumor cells tend to acquire resistance to pharmaceutical interventions. To fulfill these metabolic demands, tumor cells require increased energy and synthesis requirements, requiring redox processes 1-3. Several studies have examined the role of redox reactions in meeting tumor bioenergetic needs, facilitating processes like cell proliferation, differentiation, and metastasis 4. Reactive oxygen species (ROS) are essential to biological functions 5, 6. The disruption of redox balance in an organism due to oxidative stress typically coincides with the manifestation of disease disorders. The antioxidant pathway serves as a defense mechanism against the accumulation of ROS and contributes to the promotion of tumor growth by mitigating the detrimental effects of oxidative stress. Reactive oxygen species (ROS) are essential to biological functions. The primary cellular antioxidant enzyme systems are the thioredoxin (Trx) and glutathione (GSH) systems 7. Research has shown that inhibiting glutathione synthesis alone is effective during the initial stages of cancer. Nevertheless, the Trx system functions as a proficient compensatory antioxidant route that facilitates the advancement of tumors during the later phases of carcinogenesis 8, 9. Given the crucial role antioxidant enzymes play in cancer cells, recent studies have indicated that manipulating redox components may be beneficial in cancer treatment 10-12.

The Trx system is commonly recognized as comprising of thioredoxin (TXN, Trx), thioredoxin reductase (TXNRD, TrxR), and nicotinamide adenine dinucleotide phosphate (NADPH). Additionally, thioredoxin-interacting protein (TXNIP), an endogenous inhibitor of thioredoxin, is included. The Trx system is regulated negatively by TXNIP, which interacts with Trx and impedes its reducing activity 13. Moreover, Trx has been observed to reduce protein disulfides by acquiring reducing equivalents from NADPH through the use of TXNRD. Within the realm of tumor therapeutic research, the study of redox metabolism in tumor cells has emerged as a promising field. Through the exploration of therapeutic indications of the Trx system, valuable insights may be gained that can help patients receive a more effective cancer treatment. Previous studies have demonstrated that down-regulation of the expression of Trx, either by small molecule compounds or by genetic means, could affect the drug sensitivity of the tumor 14, 15. However, the extent to which these genes contribute to the prognosis of tumors remains uncertain.

Our investigation focuses on the assessment of the association between the Trx system and patient prognosis as a means of evaluating its immunotherapeutic relevance in order to enhance our understanding of the molecular underpinnings of the Trx system in cancer immunity. In this study, we provide a comprehensive molecular framework for the study of redox-mediated pathways in cancer that is relevant to upcoming therapeutic and progressive studies.

## Materials and Methods

### The expression and distribution of Trx system proteins

In U-251 MG and U-2 OS cells, immunofluorescence (ICC-IF) was used to study the subcellular distribution of Trx system proteins. ICC images were downloaded from the Human Protein Atlas (HPA) (http://www.proteinatlas.org/). Gene expression data in normal tissues and tumors were obtained from the Genotype-Tissue Expression database (GTEx) (https://www.gtexportal.org/home/) and The Cancer Genome Atlas Program (TCGA) database (https://portal.gdc.cancer.gov/).

To further evaluate differences in Trx system expression at the protein level, IHC images of protein expression in normal tissues and tumors tissues, including gastric cancer, lung cancer and colorectal cancer, were downloaded from the HPA databases and analyzed. Clinical Proteomic Tumor Analysis Consortium (CPTAC) databases were used to explore expression at protein level in several cancer types.

### Cell culture

Human normal lung epithelial cell line Beas-2b and human lung cancer cell lines A549, NCI-H23, NCI-H226, NCI-H838, NCI-H3122 and NCI-H1975 were obtained from the Cell Bank of the Chinese Academy of Sciences (Shanghai, China) and the American Type Culture Collection (ATCC; Manassas, VA, USA). Beas-2b cells were cultured in n BEBM Bronchial Epithelial Cell Growth Basal Medium (Lonza, CC-3171) with growth factors and supplements from BEGM Bronchial Epithelial Single Quots Kit (Lonza, CC-4175). NCI-H23, NCI-H226, NCI-H838, NCI-H3122 and NCI-H1975 cells were cultured in Roswell Park Memorial Institute (RPMI)-1640 medium, supplemented with 10% fetal bovine serum (FBS; Thermo Fisher Scientific, Waltham, MA, USA) and 1% antibiotic mixture containing 100 U/mL penicillin and 100 mg/mL streptomycin (P/S). A549 cells were cultured in Ham's F-12K medium, supplemented with 10% FBS and 1% P/S. All cell lines were maintained in a CO_2_ incubator (5% CO2) at a temperature of 37 ℃.

### Western blotting analysis

Cell lysates were harvested using a RIPA lysis and extraction buffer (Thermo Fisher Scientific, 89900) supplemented with protease and phosphatase inhibitors. The total protein concentration of the cell lysates was determined using a BCA protein quantification kit (Beyotime, P0012) according to the manufacturer's protocol. Proteins from each sample were separated on NuPAGE™ bis-tris Mini protein gels (Invitrogen, NP0336BOX) and transferred to nitrocellulose membranes (Invitrogen, IB23001). After blocking in Tris-buffered saline with Tween (TBST) buffer containing 5% w/v bovine serum albumin, the membranes were probed with primary antibodies overnight at 4 °C. The membranes were washed in TBST and incubated with secondary antibodies for 1 h at room temperature. Image acquisition and band intensity quantification were performed using a fluorescence and chemiluminescence image system (Qin Xiang, ChemiScope 6100) and Image J software (NIH, Bethesda, USA), respectively. The following primary antibodies were obtained from Cell Signaling Technology and used at a dilution of 1:1000: anti-Thioredoxin 1 (C63C6) (#2429), anti-TXNIP (D5F3E) (#14715), anti-Thioredoxin reductase 1 (TXNRD1) (#15140) and anti-GAPDH (14C10) (#2118). Band intensity quantification were performed using Image J software (NIH, Bethesda, USA).

### siRNA transfection

Gene knockdown was performed by RNA interference. Briefly, the cells were transfected with 40nM siRNA using the Lipofectamine™ 3000 Transfection Reagent kit for 48 h. knockdown efficiency was determined by western blot analysis. The siRNA sequences used are listed in Supplementary Table 1.

### Cell proliferation assay

Cells were seeded at a density of 2000-4000 cells per well in 96-well microtiter plates and subjected to various concentrations of the indicated drugs for 96h. Following the treatment, cell proliferation ability was measured using MTT assay. IC50 values were generated using GraphPad Prism.

For siRNA knockdown analysis, siRNA-transfected cells were seeded into 96-well plates and incubated for 48h, 72h, 96h and 120h at 37°C in a 5% CO_2_ incubator. MTT solution was added and incubated for 4 h. Then, the medium was withdrawn from the plates and 200 µl DMSO was added to each well to solubilize the formazan crystals. The absorbance was detected at 570 nm.

### The relationship between Trx system and prognosis

The Kaplan-Meier plotter database (http://kmplot.com/analysis/) were used to explore the association between Trx system and survival outcome of patients in pan-caner. Patients were divided into a high-risk group (high expression of TXN and TXNRD and low expression of TXNIP) and a low-risk group (low expression of TXN and TXNRD and high expression of TXNIP) for analysis. The receiver operating characteristic (ROC) curve was used to assess the diagnostic value of Trx system in pan-cancer via R package “pROC”. The area under the ROC curve (AUC) was applied to evaluate the predictive performances of the test biomarkers.

### GO and KEGG analysis of Trx system

Gene Ontology (GO) and Kyoto Encyclopedia of Genes and Genome (KEGG) were used via the R package “clusterProfiler” to analyze potential pathway related to DEGs between low and high expression gene sets, and visualized with the OmicShare tool (https://www.omicsmart.com/), an online platform for data analysis. When p < 0.05 and FDR of < 0.1, the gene sets were deemed reliable.

### Analysis of the correlation between Trx system and tumor immunity

The major hallmarks of the tumor microenvironment are associated with tumor progression and recurrence. To predict the level of immune infiltration, ESTIMATE (Estimation of Stromal and Immune cells in MAlignant Tumor tissues using Expression data) method was used for the assess the stromal and immune cells in tumor tissues 16. We also used ssGSEA to estimate the fraction levels of immune-related gene sets for each cancer sample in the subgroup 17. The single-cell analysis was conducted online in Tumor Immune Single-cell Hub (TISCH) database (http://tisch.comp-genomics.org/home/).

### Analysis of the correlation between Trx system and immunotherapy response

Tumor Immune Dysfunction and Exclusion (TIDE) is a computational framework developed to evaluate the potential of tumor immune escape from the gene expression profiles of cancer samples 18, 19. We explored the relationship between the Trx system and T cell dysregulation and its relevance to immunotherapy clinical response. The comparison between Trx system and other published biomarkers based on their predictive power of response outcome and overall survival was performed with TIDE.

Tumor Immune Syngeneic Mouse (TISMO) is a database, which contains includes 1518 *in vivo* RNA-seq samples from 68 syngeneic mouse tumor models across 19 cancer types, of which 832 were from immune checkpoint blockade (ICB) studies 20. We used this database to investigate the correlation between the Trx system and differential immunotherapy response.

### Statistical analysis

The Wilcoxon rank sum test was applied to compare normal and cancer tissue, and the difference among variables. All survival analyses in this investigation used the Kaplan-Meier curve. The “ggplot2”, “pheatmap”, packages in R software (https://www.r-project.org/) were used for visualization. The MTT results were presented as mean ± SD. Statistical analysis was conducted using GraphPad Prism 9.0 software.

## Results

### The expression profile of the Trx system in humans

**Figure 1:**
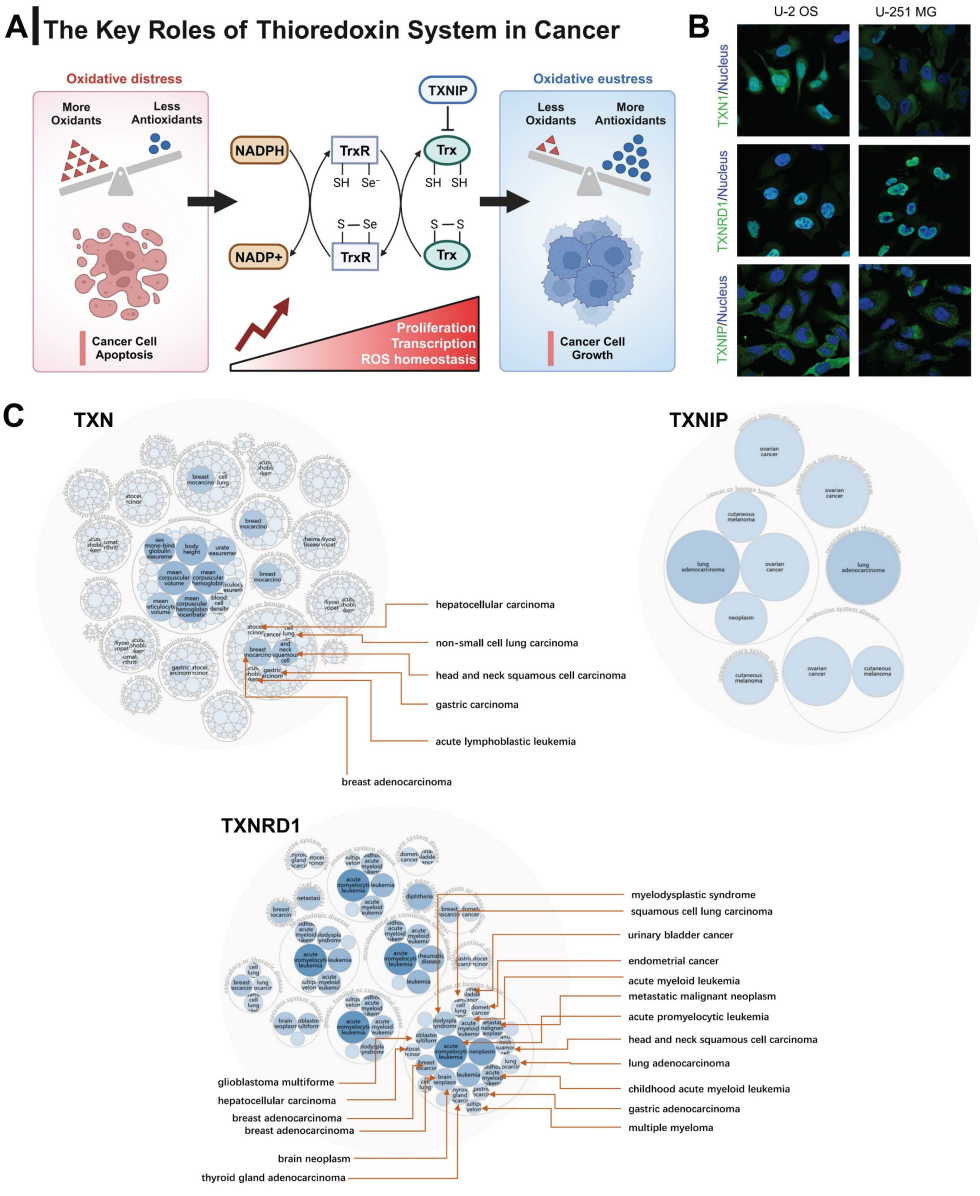
The expression profile of the Trx system in humans. (A) The demonstration of the basic functions of the Trx system in cancer. (B) The distribution of the subcellular localization of Trx system proteins in U-251 MG and U-2 OS cells by the Human Protein Atlas (HPA) database. (C) Disease network interaction analysis between various tumors and Trx system genes.

The occurrence of disease is often accompanied by a disruption of redox equilibrium inside the organism 21-23. The Trx system, which plays a significant role in maintaining redox homeostasis, has been implicated in the pathogenesis of several diseases, including cancer, neurological disorders, diabetes, and cardiovascular diseases 24-26. Breg cell differentiation relies on homeostatic levels of ROS. TXN insufficiency as a cause of Breg cell impairment in patients with systemic lupus erythematosus (SLE) 27. TXN plays a neuroprotective role in Parkinson disease by suppressing ER stress and inhibiting IRE1 activation 28, 29. TXNIP activates NLRP3 inflammasome complex formation, triggers mitochondrial stress-induced apoptosis and inflammatory cell death, as a potential therapeutic target in various diseases, such as diabetes, chronic kidney disease, and neurodegenerative diseases 30. Inhibiting TXNRD1 maintains TXN in its oxidized state, restricting Caspase-11 activation and alleviating sepsis 31. Figure 1A illustrates the primary functions of the Trx system in the context of cancer. The Trx system plays a key role in redox regulation and is involved in a variety of physiological functions, including tumor growth, immune response, and stem cell differentiation.

In order to analyze the subcellular localization of genes related to the Trx system, we initially examined the distribution patterns of TXN, TXNIP, and TXNRD1 in U-251 MG and U-2 OS cells by utilizing the Human Protein Atlas (HPA) database. The co-localization of TXNRD1 with the nuclear marker in U-251 MG and U-2 OS cells was observed, indicating the subcellular localization of TXNRD1 within the nuclei. In contrast, the nuclear localization of TXNIP was not seen in U-251 MG and U-2 OS cells. TXN predominantly exhibits localization within the nucleoplasm and cytosol (Figure 1B). The occurrence and development of various types of malignancies have been significantly linked to all three genes through disease network interaction analysis (Figure 1C). We also investigated the mRNA expression levels of TXN, TXNIP, and TXNRD1 in different normal human tissues obtained from the GTEx database. These tissues included numerous systems such as the immunological, neurological, muscular, internal, secretory, and reproductive systems (Figure S1).

### The expression of the Trx system in pan-cancer

**Figure 2:**
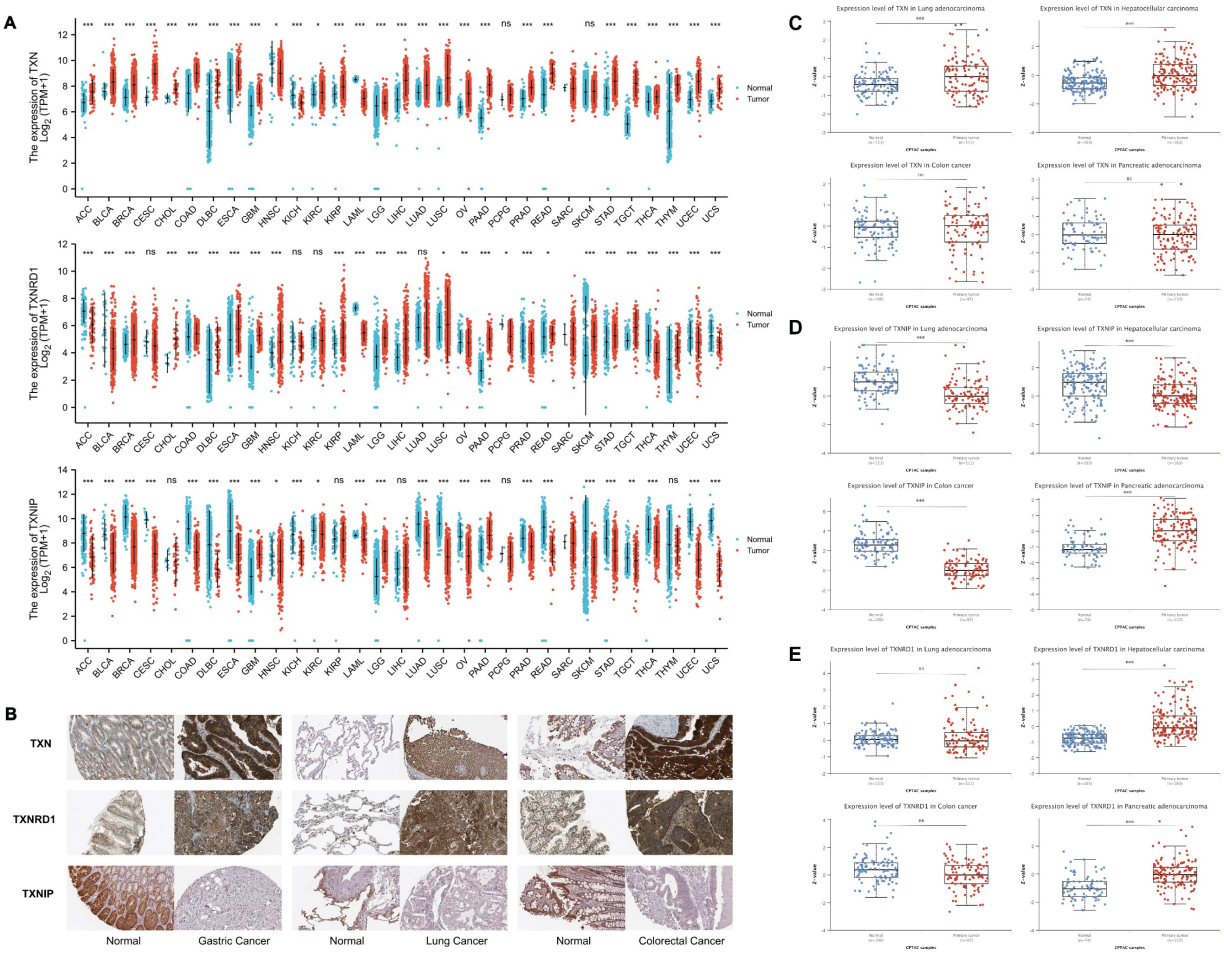
The expression of the Trx system in pan-cancer. (A) The expression of selected genes in pan-cancer based on TCGA and GTEx database. (B) Immunohistochemical images of Trx system proteins in tumor and normal tissues of gastric cancer, lung cancer and colorectal cancer from HPA database. (C) The protein expression levels of TXN, TXNIP and TXNRD1 based on CPTAC database.

We first evaluated the expression of TXN, TXNRD1, and TXNIP in pan-cancer data from TCGA and GTEx to further investigate the connection between the Trx system and malignancies. ACC, BLCA, BRCA, CESC, CHOL, COAD, DLBC, ESCA, GBM, KIRC, KIRP, LGG, LIHC, LUAD, LUSC, OV, PAAD, PRAD, READ, STAD, TGCT, THCA, THYM, UCEC, and UCS were among the cancers with high TXN expression, according to the analysis. TXNRD1 showed higher expression levels in BRCA, COAD, CHOL, DLBC, ESCA, GBM, HNSC, KIRP, LGG, LIHC, LUSC, PAAD, READ, SKCM, STAD, TGCT, and THYM. ACC, BLCA, BRCA, CESC, DLBC, ESCA, HNSC, KICH, KIRC, LAML, LUAD, LUSC, OV, PRAD, READ, SKCM, STAD, TGCT, THCA, UCEC, and UCS, on the other hand, had lower levels of TXNIP expression (Figure 2A). Significant differences were found in all three indicators in these cancer types including BRCA, COAD, DLBC, ESCA, LUSC, READ, STAD and TGCT. In the remaining cancer types, one or two of these three genes had abnormal expression. Using TCGA datasets, we then explore the mRNA levels of TXN, TXNRD1, and TXNIP in tumor and adjacent normal tissues and found similar results (Figure S2).

In order to examine the expression of the Trx system protein in normal and malignant tissues, we got immunohistochemistry images from the Human Protein Atlas (HPA) (Figure 2B). TXN and TXNRD1 expression levels significantly increased in tumor tissue samples from colorectal, lung, and stomach cancers. In contrast, normal tissues showed a more prominent expression of TXNIP. Using the CPTAC database, we verified that TXN, TXNRD1 and TXNIP proteins were also aberrantly expressed in the tumor (Figure 2C-E).

### Oncogenic function of the Trx system in lung cancer

Recently, studies have reported the role of the Trx system in cancer development and drug resistance. Co-targeting of BTK and TXNRD1 is an effective therapeutic strategy to consider for Diffuse large B-cell lymphoma (DLBCL) treatment 32. IL-6-induced nuclear translocation of the TXN-pSTAT3 complex is closely correlated with lymph node metastasis and distant metastasis in CRC 33.

**Figure 3:**
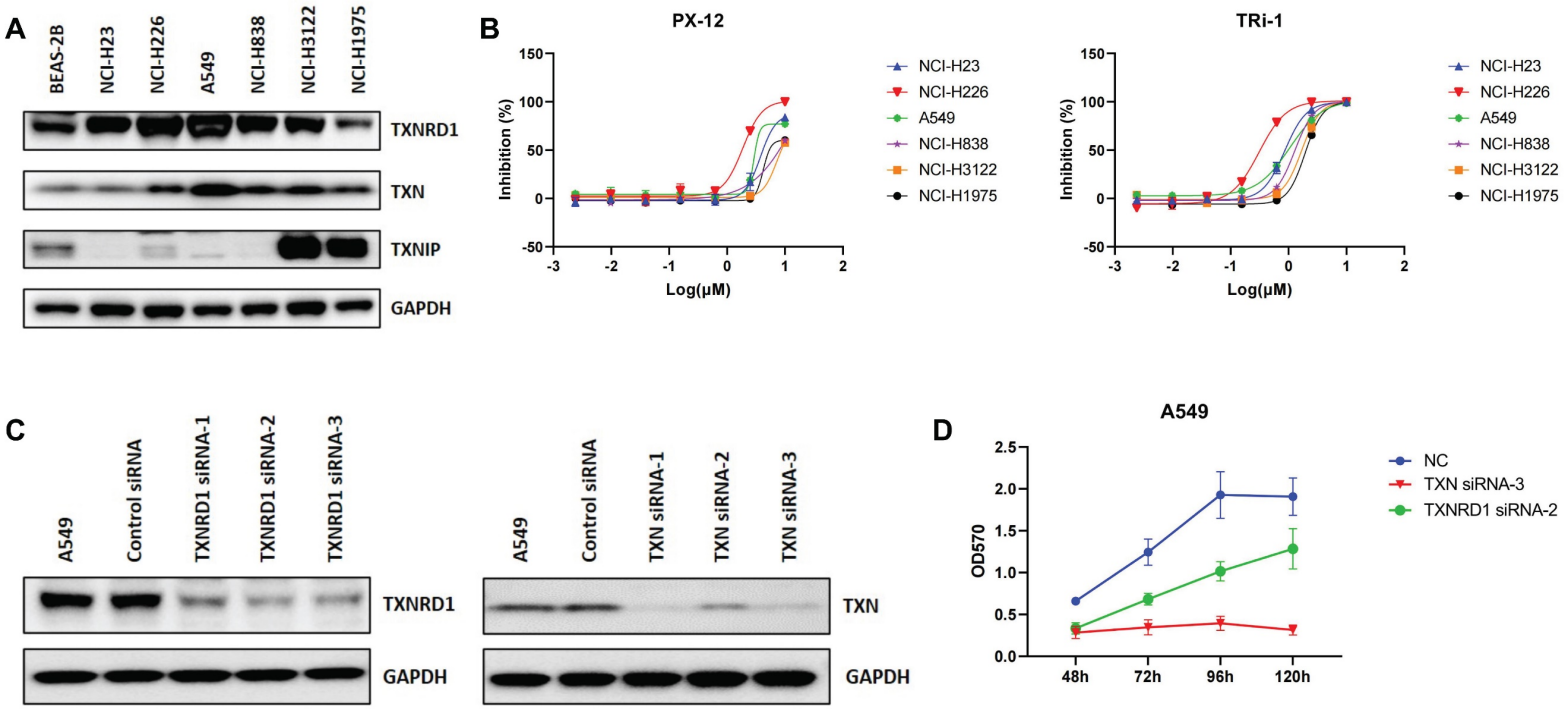
The expression of the Trx system in lung cancer. (A) The protein expression levels of TXN, TXNIP and TXNRD1 in cell lines with western blot analysis. (B) Cell proliferation was assessed by MTT assay after treatment of A549 cells with PX-12 or TRi-1 for 4 days. (C) Western blot analysis of protein expression in A549 transfected with TXN-siRNA or TXNRD1-siRNA. (D) Cell proliferation was assessed by MTT assay in A549 following transfection with TXN-siRNA or TXNRD1-siRNA.

Based on the above analysis, we verified the expression of the Trx system in lung cancer cell lines by western blot analysis (Figure 3A). The results showed that the expression of TXN and TXNRD1 was higher in most lung cancer cell lines than in the normal lung epithelial cell line Beas-2b, while the opposite was observed for TXNIP. PX-12, a TXN-specific inhibitor that has been evaluated in phase I trials in several advanced metastatic cancers, irreversibly binds to TXN protein and render it redox inactive 34. Both inhibition of Trx by PX-12 and overexpression of TXNIP restored sensitivity to chemotherapeutic drug 35. TRi-1 is a novel and irreversible inhibitor of TXNRD1, showing anticancer efficacy with low mitochondrial toxicity 36, 37. The MTT assay revealed anticancer potential of PX-12 against lung cancer A549, NCI-H23, NCI-H226, NCI-H838, NCI-H3122 and NCI-H1975 cells, with the half inhibitory concentration (IC50) values were 5.3 µM, 4.9 µM, 1.8 µM, 7.7 µM, 8.9 µM, and 9.6 µM, respectively. IC50 values of TRi-1 in A549, NCI-H23, NCI-H226, NCI-H838, NCI-H3122 and NCI-H1975 cells were 0.96 µM, 0.87 µM, 0.33 µM, 1.3 µM, 1.7 µM, and 2.3 µM, respectively (Figure 3B).

To further investigate the role of the Trx system in cancer, we used small interfering RNAs (siRNAs) to knock down TXN and TXNRD1 in lung cancer cell lines. The expression of TXN and TXNRD1 was significantly decreased (Figure 3C). Therefore, TXN siRNA-3 and TXNRD1 siRNA-2 were used to knockdown TXN and TXNRD1 expression. We observed significant growth inhibition upon siRNA-mediated TXN or TNRD1 knockdown in A549 (Figure 3D). siRNA-mediated knockdown of TXN and TXNRD1 inhibited cell proliferation of lung cancer cells. In our study, we found that disrupting the balance of the Trx system, whether using siRNAs or inhibitors, was able to inhibit tumor cell proliferation, indicating that Trx system may play an important role in cancer.

### Survival analysis of Trx system in pan-cancer

**Figure 4:**
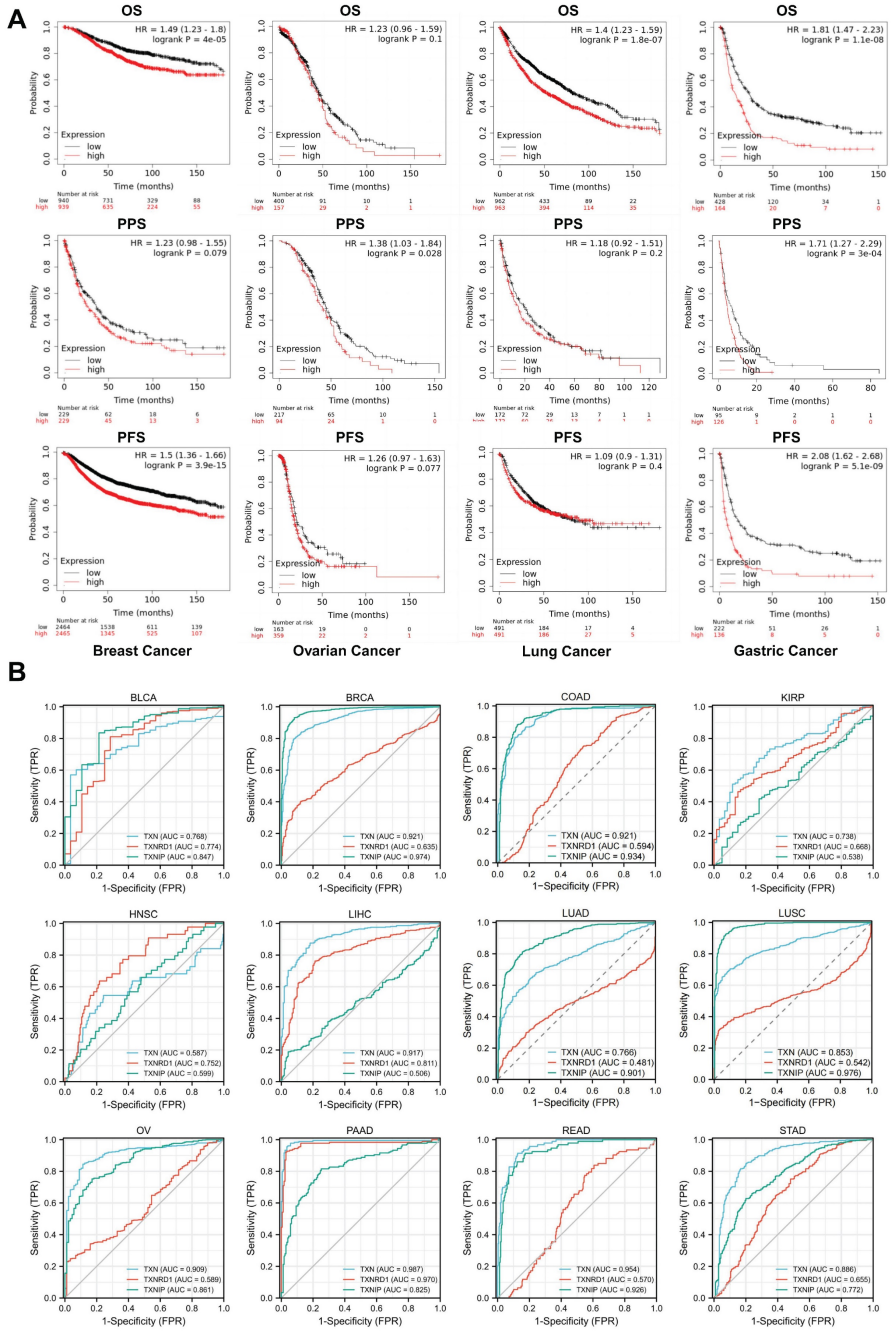
Survival analysis of Trx system in pan-cancer (A)The Kaplan-Meier survival curve of Trx system genes in pan-cancer. (B) The receiver operating characteristic (ROC) of Trx system genes in pan-cancer.

The Kaplan-Meier survival curve study of the prognostic importance of Trx system expression was based on the Kaplan-Meier plotter database to assess the utility of Trx system genes in predicting the prognosis of cancer patients. We identified low expression of TXNIP and high expression of TXN and TXNRD1 as high-risk variables. Aberrant expression of TXN, TXNRD1 and TXNIP was significantly correlated with overall survival (OS), post-progression survival (PPS) or progression-free survival (PFS) (Figure 4A). We further confirmed the associations between Trx system and prognosis in pan-cancer by analyzing the expression of Trx system with overall survival (OS) and relapse-free survival (RFS) in malignancies of the bladder, head and neck, kidney, liver, and lung (Figure S3).

The diagnostic utility of Trx system genes in pan-cancer was evaluated using the receiver operating characteristic (ROC) curve. The findings demonstrated that the Trx system was capable of accurately predicting 12 different cancer types. TXN had a high accuracy (AUC > 0.9) in predicting BRCA, COAD, LIHC, OV, PAAD and READ, and TXNIP had a high accuracy (AUC > 0.9) in predicting BRCA, COAD, LUAD, LUSC and READ (Figure 4B). The results of our study suggest that the Trx system, which consists of TXN, TXNRD1, and TXNIP, may be useful for the early diagnosis and monitoring of cancer.

### The Trx system is implicated in the regulation of processes involved in metabolism and tumor immunity

**Figure 5:**
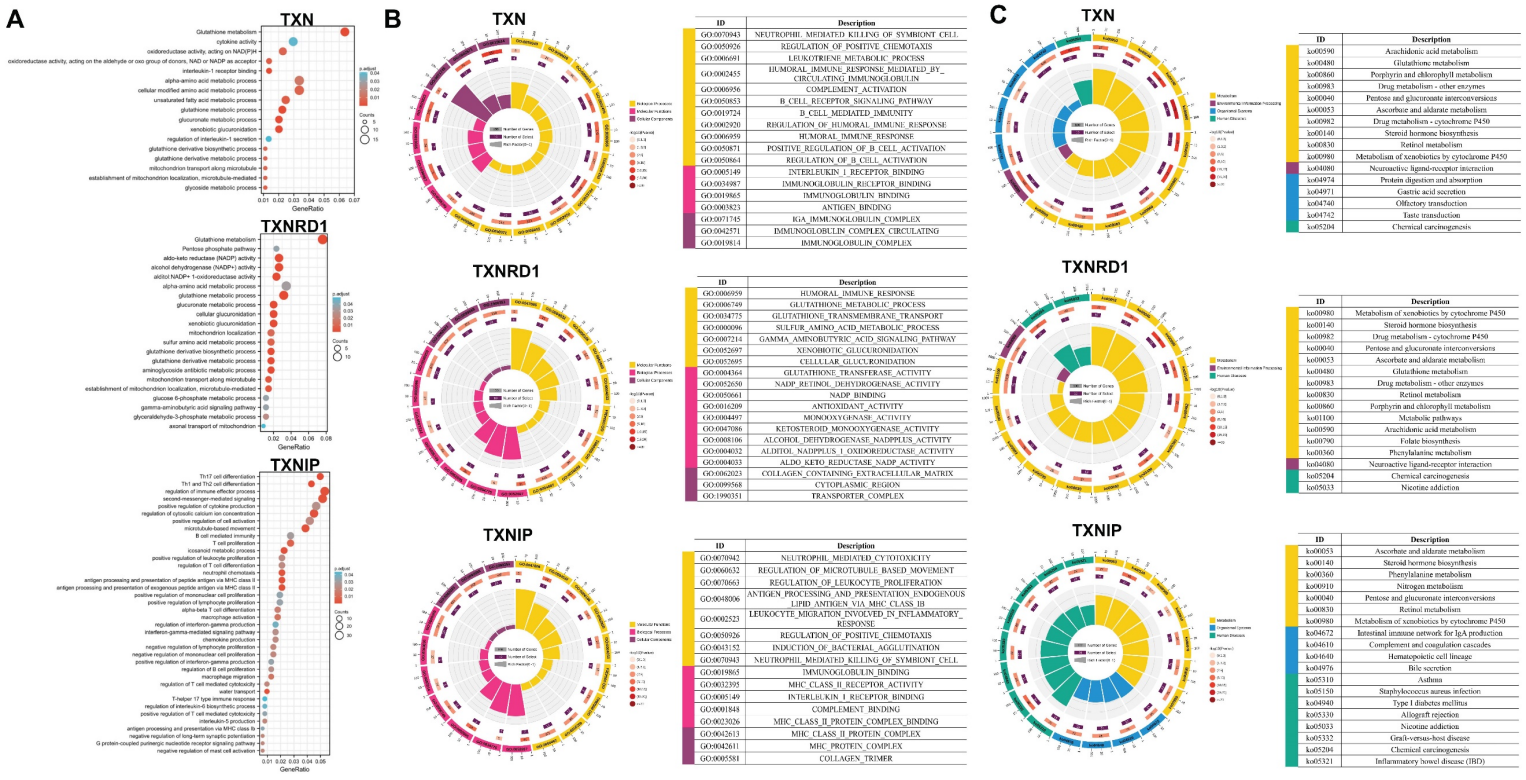
Enrichment Analysis of the Trx system (A-B) The enrichment circle of GO (A) and KEGG (B) analysis of Trx system genes in LUAD and LUSC. The first circle represents the top terms, and the number of genes corresponds to the outer circle. The second circle shows the number of genes in the enriching specific terms. The third circle shows the number of the upregulated and downregulated genes. The fourth circle represents the enrichment factor of each term.

TXN/TXNRD1 inhibitors not only have anti-tumor activity, but also inhibit pulmonary fibrosis by inhibiting NF-κB/TGF-β1/Smads pathway 38. Inhibition of TXN resulted in blockage of the glycolytic pathway and significantly increased the cytotoxicity of 2DG on CRC cells 39. The ATM/AKT pathway is a compensatory mechanism to cope with the ROS accumulation induced by the inhibition of the Trx system in colon cancer 40. We performed gene set enrichment analysis of the Trx system, including the enrichment of signaling pathways by GO and KEGG in lung cancer and gastric cancer (Figure 5 and Figure S4), to examine the effect of the Trx system on malignancies.

TXN and TXNRD1 were both strongly associated with metabolism, including glutathione and amino acid metabolisms. They also involved processes involving NADPH production and consumption routes, as well as glucose metabolism. In contrast to TXN and TXNRD1, TXNIP was found to be strongly associated with immunity, including immune activation and response, immune cell proliferation and differentiation, and cytokines. These findings reveal that signaling pathways involved in tumor metabolism and tumor immunity are closely associated with the Trx system.

### The Trx system is associated with tumor immune

The immune-inflammatory response and the redox system are closely related processes 41. The Trx system plays an essential role in the initiation of immune reactions and regulation of inflammatory responses during bacterial infections 42. By controlling intracellular and extracellular ROS levels, the Trx system regulates the survival and performance of immune cells, including immune cell polarization, immune tolerance, metabolic reprogramming, cytokines production, and chemotaxis 43, 44. In addition to being employed as a therapeutic target for targeted therapy, it can also be used in the modification of immunotherapeutic tools. Genetic human Trx1-transgenic (Trx1-Tg) donor T cells or administration of human recombinant Trx1 to recipients of allogeneic hematopoietic transplantation (allo-HCT) could both considerably lessen the severity of graft-versus-host disease (GVHD) 45.

**Figure 6:**
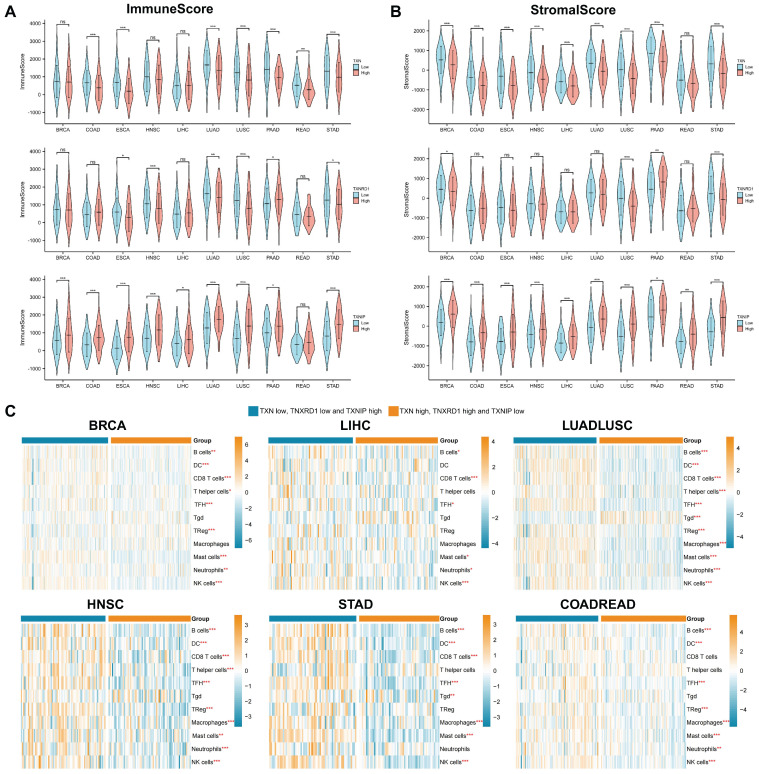
The association between the Trx system and tumor immune. (A-B) Violin diagrams depicted the relationship between Trx system and the immune (A) and stromal (B) scores in pan-cancer. (C) Heatmaps demonstrated the association between the Trx system and tumor infiltrating immune cells (TIICs) based on ssGSEA in pan-cancer.

By analyzing the genes and signaling pathways co-expressed with the Trx system, we discovered that it was highly associated with metabolism and tumor immunity, so we focused on tumor immunity in the subsequent analysis. The stromal and immune scores of the pan-cancer were assessed first in order to understand the relationship between Trx and the tumor immune microenvironment. Significant variations between the high and low expression groups were seen in the stromal and immune scores. In most cancer species, the TXN and TXNRD1 high expression groups and the TXNIP low expression group had lower stromal and immune scores (Figure 6A-6B).

Recent work has identified that the Trx system plays an important role in modulating signaling between different cell types within tumor microenvironment 46. Tumor-infiltrating immune cells have important implications for tumor treatment outcomes and patient prognosis. Using ssGSEA, we examined the relationship between the Trx system and tumor infiltrating immune cells (TIICs) in various cancer type. (Figure 6C). Immune cell expression varied in statistically significant ways between the groups. The high expression of TXN and TXNRD1, as well as the low expression of TXNIP group exhibited lower levels of tumor immune infiltration. However, the low expression of TXN and TXNRD1, and high expression of TXNIP group was enriched in many kinds of immune cells, especially B cells, follicular helper T cell (TFH), mast cells and NK cells. To further investigate the relationship between Trx system and tumor immunity, we obtained the mRNA expression level of TXN, TXNRD1 and TXNIP at single-cell level using 10 datasets from TISCH data. The results illustrated Trx system genes is widely expressed in immune cells, especially T cells (Figure S5). These findings demonstrated that a deactivated immune microenvironment could be facilitated by an aberrant redox system.

### The Trx system is associated with T cell dysfunction

Numerous studies have demonstrated a significant correlation between TXNIP and immunity and inflammation. It is consistent with the findings of the enrichment pathway analysis discussed above. TXNIP, as well as TXN and TXNRD1 have been demonstrated in several studies to regulate tumor immunity, especially T-cell function and differentiation 47. TXN may suppress T-cell antitumor responses and favor metastatic melanoma by recruiting Treg cells 48. TXNRD1 inhibitor can cause immunogenic cell death (ICD) *in vivo*, encourage DC maturation, and elicit T-cell responses, all of which lead to tumor regression 49.

**Figure 7:**
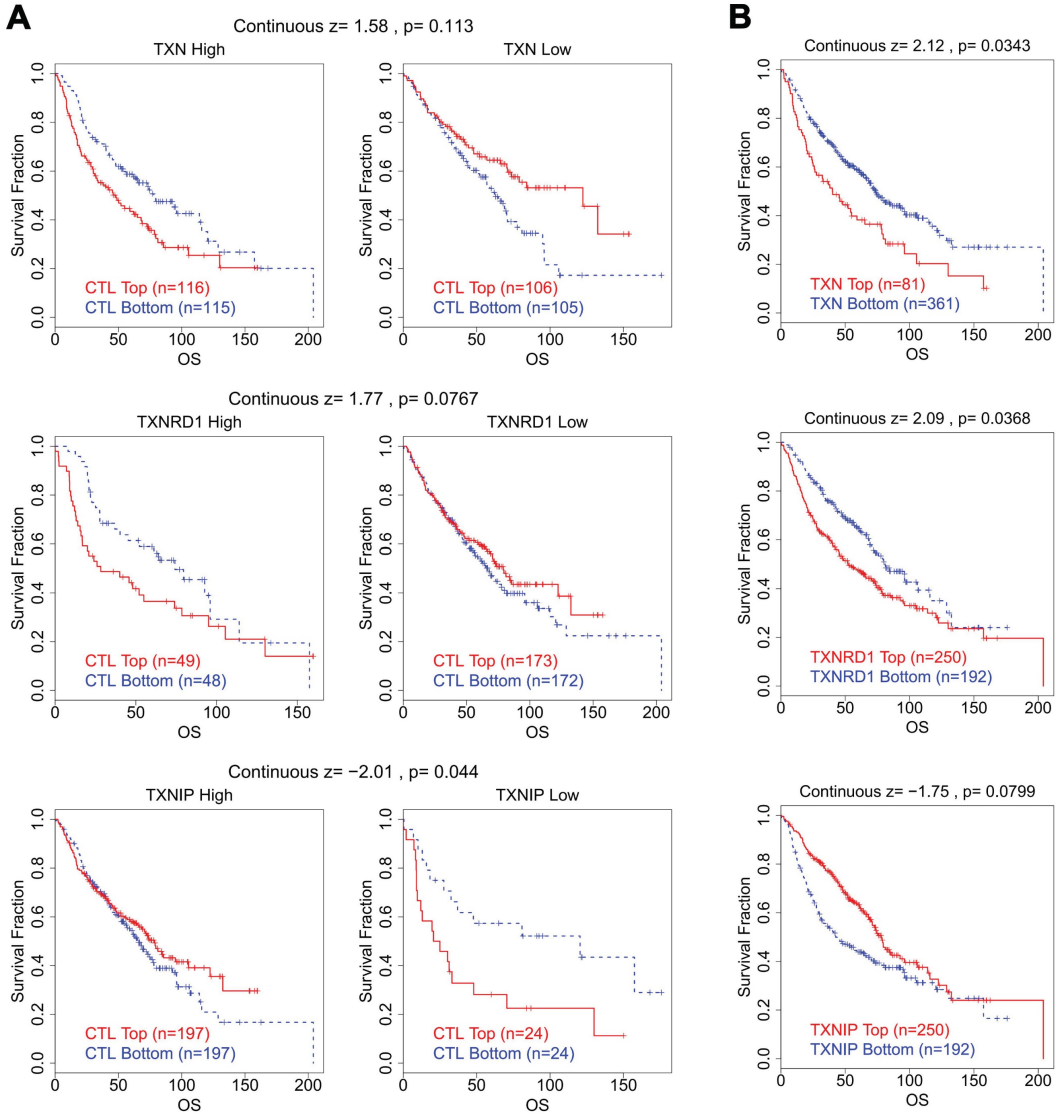
The Correlation Analysis between Trx System and T Cell Dysfunction in lung cancer. (A) The survival curves illustrated the relationship between dysfunctional T cell phenotype and prognosis in different expression levels of Trx system. (B) The survival curves illustrated the association between the expression of Trx system genes and prognosis in dysfunctional T cell phenotype.

We looked studied the connection between CTL levels and OS of patients in various groups with varying expression levels of the Trx system in lung cancer in order to better understand whether there is a clinical correlation between abnormal expression of Trx system and T cell dysfunction. Intriguingly, we discovered that lung cancer patients with high levels of TXN and TXNRD1 expression had shorter survival duration and dysfunctional T cell phenotype (Figure 7). In contrast, in the same group of patients, increased expression of TXNIP was connected to a favorable prognosis with normal T cell phenotype.

High expression of TXN and TXNRD1 with low expression of TXNIP was associated with dysfunctional T cell phenotype and shorter survival of the breast carcinoma cohort (Figure S6) and colorectal carcinoma cohort (Figure S7), in accordance with the abnormal expression status of the Trx system in BRCA and COAD.

### Response to immune checkpoint blockade therapies is associated with the Trx system

**Figure 8:**
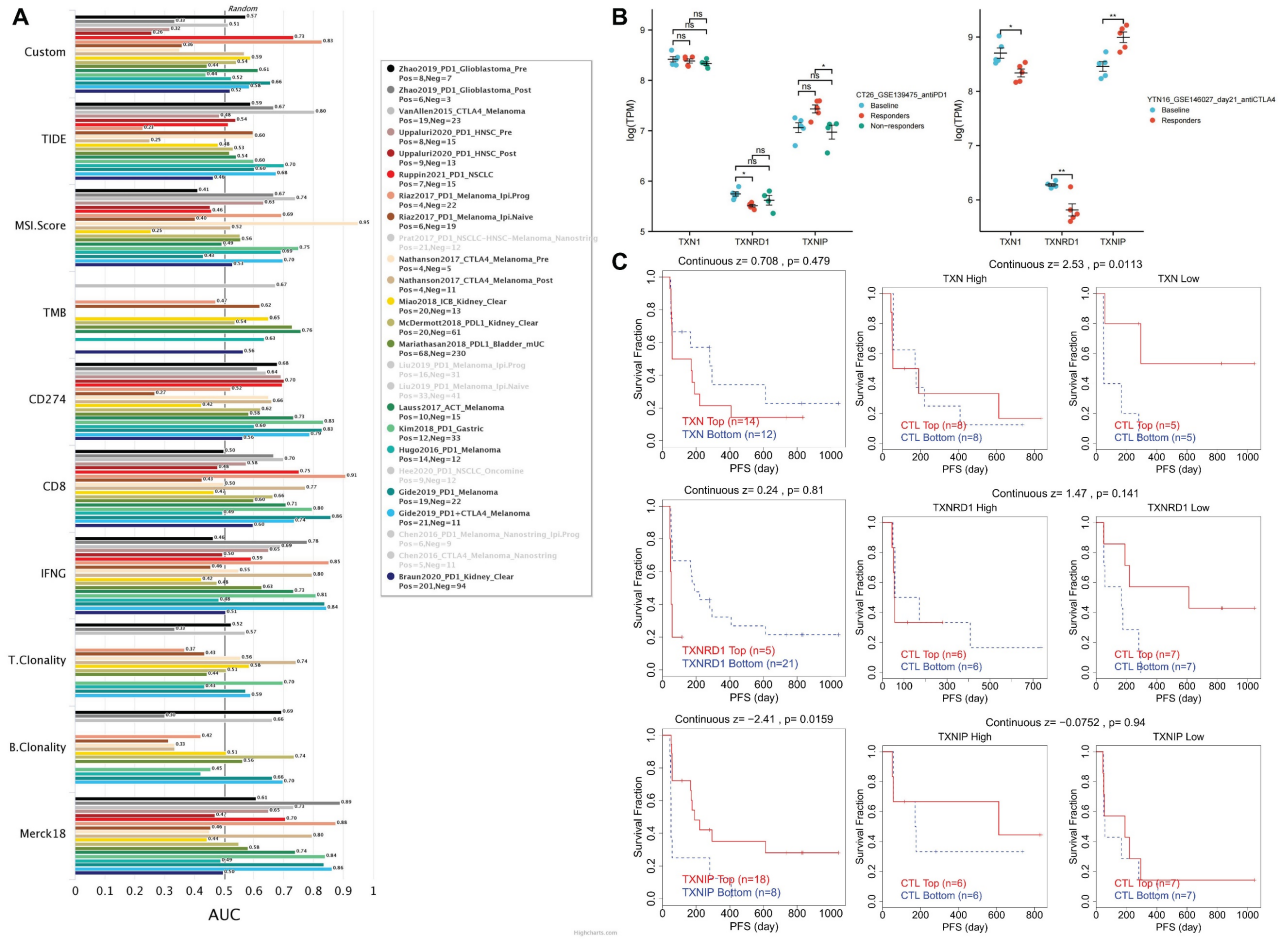
Assessment of the correlation of Immune Checkpoint Blockade Therapies in the Trx system. (A) The AUC values of the ICB sub-cohorts containing the new biomarker constructed with three genes. (B) The relationship between Trx system expression and treatment response in ICB-treated mouse model based on the TISMO database. (C) The correlation analysis between Trx system and T cell dysfunction in the Riza2017_PD1 sub-cohort.

By contrasting the Trx system with standardized biomarkers used to forecast response outcomes and OS of ICB sub-cohorts, the biomarker relevance of the system was assessed. In 23 ICB sub-cohorts, we combined high expression of TXN and TXNRD1 with low expression of TXNIP to create a new biomarker, and we compared it to existing biomarkers. The three genes were mapped in 17 ICB sub-cohorts, and had an area under the receiver operating characteristic curve (AUC) of > 0.5 in 12 of the 17 ICB sub-cohorts (Figure 8A). The Trx system exhibited a higher predictive value than TMB, T. Clonality, and B. Clonality and was comparable to the MSI score (AUC > 0.5 in 12 ICB sub-cohorts), but lower than TIDE, CD274, CD8, IFNG, and Merck18.

Intriguingly, we discovered that T cell dysfunction on tumor ICB-treatment sub-cohorts is also correlated with the Trx system. Using the TISMO database, we first confirmed the relationships between Trx system expression and treatment response in the ICB-treated mouse model (Figure 8B). We noticed a rise in TXNIP and a fall in TXNRD1 for responders in the anti-PD1-treated CT26 mouse model. TXNRD1 levels were noticeably lower in responders in the anti-CTLA4-treated YTN16 mouse model. The high expression of TXN and TXNRD1 groups, as well as the low expression of TXNIP group, were shown to be accompanied by T cell dysregulation and shorter survival in the Riza2017_PD1 sub-cohort (Figure 8C). Other ICB sub-cohorts showed similar results (Figure S8).

## Discussion

Inactivation of tumor suppressors and activation of oncogenes both lead to the production of cancer cells. Unrestricted growth is a crucial feature of cancer cells and is associated by high levels of ROS generation. As a result, cancer cells must protect themselves from the deleterious effects of oxidative stress by developing an antioxidant system that keeps ROS at a level that does not interfere with tumor growth 50, 51. In multiple clinical trials, increased tumor risk has also been linked to the consumption of antioxidant supplements, and targeting antioxidant pathways offers new approaches to both the prevention and treatment of cancer 52. Large amounts of energy are needed for the aberrant growth and metastasis of cancer cells, which is accompanied by high levels of ROS production. Antioxidant systems are necessary to buffer cancer cells from high ROS levels that cause cellular damage 53. Targeting antioxidant-associated proteins in cancer cells can prevent cancer cell proliferation, tumorigenesis, and metastasis 54.

The Trx system is crucial to the redox system, particularly in unique physiological contexts like tumors and inflammation 30, 55. The increasing number of studies that are focused on TXN, TXNRD1, and TXNIP for the purpose of cancer therapy has further demonstrated the importance of the Trx system in the development of cancer Several studies have shown that the disruption of the Trx system can lead to increased oxidative stress, inflammation, and apoptosis, which are all associated with cancer progression. Therefore, understanding the Trx system is important for the development of new cancer treatments 56-59. The Trx system is also involved in the regulation of inflammation and autoimmune diseases, and has been shown to be a determinant of sensitivity to Checkpoint kinase 1 inhibitors 60. This is in line with the acknowledgement of the importance of the Trx system. It is unclear how the expression patterns of genes of Trx system and prognostic diagnoses relate to one another.

The Trx system, as an important regulatory element of redox, is usually studied with TXN and TXNRD1 in it. In this paper, TXN, TXNRD1 and TXNIP are integrated for the first time to comprehensively analyze the importance and indication of the Trx system in tumor and immunotherapy. Here, we have done a comprehensive and holistic analysis of Trx system. We used multiple databases, including TCGA, Genotype Tissue-Expression (GTEx), and Human Protein Atlas (HPA), to analyze Trx system expression levels and their relationship with prognosis in pan-cancer. Through data analysis, we verified that gene expression of the Trx system is aberrantly expressed in tumor patients and correlates with poor prognosis. Inhibiting thioredoxin (TRX) with PX-12 could blunt the protective effect of silencing TXNIP in hypertension, suggesting that PX-12 disrupted the balance of the Trx system 61. The results of MTT showed that knocking down TXN and TXNRD1 significantly inhibited cell proliferation in A549 cells. Therefore, genetic depletion of TXN and TXNDR1 with siRNA oligonucleotides disrupts the redox balance mediated by the Trx system in tumor cells, thereby contributing to the inhibition of cell proliferation.

Metabolic abnormalities are one of the hallmarks of malignancy. The Warburg effect, or aerobic glycolysis, is the key distinction between benign cells and malignant tumor cells. Tumor cells are mainly supplied with energy through the glycolytic pathway, which enhances the ability of cells to evade apoptosis, invasion, and resistance to chemotherapeutic agents. Several reports have demonstrated that increases in TXN and TXNRD1 under glucose deprivation conditions support cancer cell survival and migration 62, 63, whereas enhanced stability of TXNIP mRNA inhibits tumor metastasis and glycolysis 64. In this paper, we further validated the correlation between the Trx system and metabolism by GO and KEGG analysis. We also found that the Trx system showed a strong correlation with immunity in this part of the analysis, which is consistent with some of the recent studies reported 65-67. The majority of immune-related studies concentrated on TXNIP and immune vesicles, however using immune correlation statistical analysis, we discovered that TXN and TXNRD1 were also strongly correlated with immune infiltration. Additionally, it showed a high consistency with patient prognosis and was closely related to T-cell dysfunction.

Immunotherapy, including immune checkpoint inhibitors and cellular immunotherapy, has emerged as a powerful clinical therapeutic strategy in tumor treatment. Redox signaling pathways influence tumor immunity by regulating the oxidative stress within tumor cells, which can affect their growth and survival. These pathways modulate the function of immune cells, such as T cells and macrophages, enhancing their ability to recognize and attack cancer cells. Redox signaling pathways play an important role in tumor immunity, and inhibitors of the Trx system have also been shown to play a role in antitumor immunity. Consequently, targeting redox signaling pathways could be a promising strategy to improve the efficacy of immunotherapy. Additionally, combining immunotherapy with inhibitors of the Redox system could be a promising approach. Redox signaling pathways play an important role in tumor immunity, and inhibitors of the Trx system have also been shown to play a role in antitumor immunity. For example, a clinical trial involving TXNRD1 inhibitor auranofin has been enrolled in phase I/II clinical trials to treat chronic lymphocytic leukemia, non-small cell lung cancer, and ovarian, peritoneal, and fallopian tube cancers (www.clinicaltrials.gov, trial numbers NCT01419691, NCT01737502, NCT01747798, and NCT03456700). These examples highlight the potential of incorporating redox pathway inhibitors in cancer treatment to improve patient outcomes 68-70. We evaluated the stromal and immune cells in tumor tissues using the ESTIMATE method, and found that stromal and immune scores were lower in the high TXN, high TXNRD1, and low TXNIP groups in most tumor species. We also used CIBERSORT as an analytical tool to provide estimates of enrichment scores for each type of immune cell in the subpopulations. Clinical outcome and immune cell infiltration in malignancies are closely connected. Immune cell infiltration and the prognosis that goes along with it are both closely tied to abnormal Trx system expression. T cells play a crucial role in tumor immunity by directly attacking and killing cancer cells. They recognize tumor-associated antigens presented on the surface of cancer cells and initiate an immune response. By enhancing the function and activity of T cells, redox signaling pathways can improve the effectiveness of immunotherapies in targeting and eliminating tumors 71, 72. Our study showed that high expression of TXN and TXNRD1 with low expression of TXNIP was associated with dysfunctional T cell phenotype. There was also a correlation between the Trx system and immunotherapy response. Based on these findings, we discovered that the Trx system is anticipated to be a good immunotherapy response predictor through the TIDE and TISMO databases.

There are also certain limitations to our study. Immunotherapy has been shown to be more effective in treating cancer, especially when combined with other treatments. Further investigation is necessary to determine whether the Trx system can accurately predict immunotherapy sensitivity and even combination therapies. Additionally, there is a need for more research to determine whether this prognostic model will be applicable to clinical practice.

## Conclusion

The Trx system shows similarities across cancer types, reflecting the importance of redox system regulation in pan-cancer. Understanding the expression of the Trx system in tumor and the tumor immune microenvironment is crucial for the development of effective cancer treatment strategies. A comprehensive analysis of the genes that constitute the Trx system is presented in this paper. We found that these genes, in addition to being effective therapeutic targets, may serve as prospective biomarkers of clinical prognosis and ICI response.

## Supplementary Material

Supplementary figures and table.
