# Supplementary material for: Exploring the Role of Thioredoxin system in Cancer Immunotherapy
Source: J Cancer. 2025 Jan 1;16(1):66–80. doi: 10.7150/jca.98306 (PMC11660121; doi:10.7150/jca.98306)
Supplement: Supplementary file 1 — Supplementary figures and table. [file jcav16p0066s1.pdf]

	Sequence (5' -> 3')
TXN siRNA-1	GCAGGUGAUAAACUUGUAGUA
TXN siRNA-2	GAUGUGGAUGACUGUCAGGAU
TXN siRNA-3	GCUUCAGAGUGUGAAGUCAAA

	Sequence (5' -> 3')
TXNRD1 siRNA-1	CCUGCAAGACUCUCGAAAUUA
TXNRD1 siRNA-2	CGUCAAGAGAUAACAACAAAU
TXNRD1 siRNA-3	GCUGGAUUUCUUGCUGGUAUU

Supplementary Table 1. siRNA sequence information.

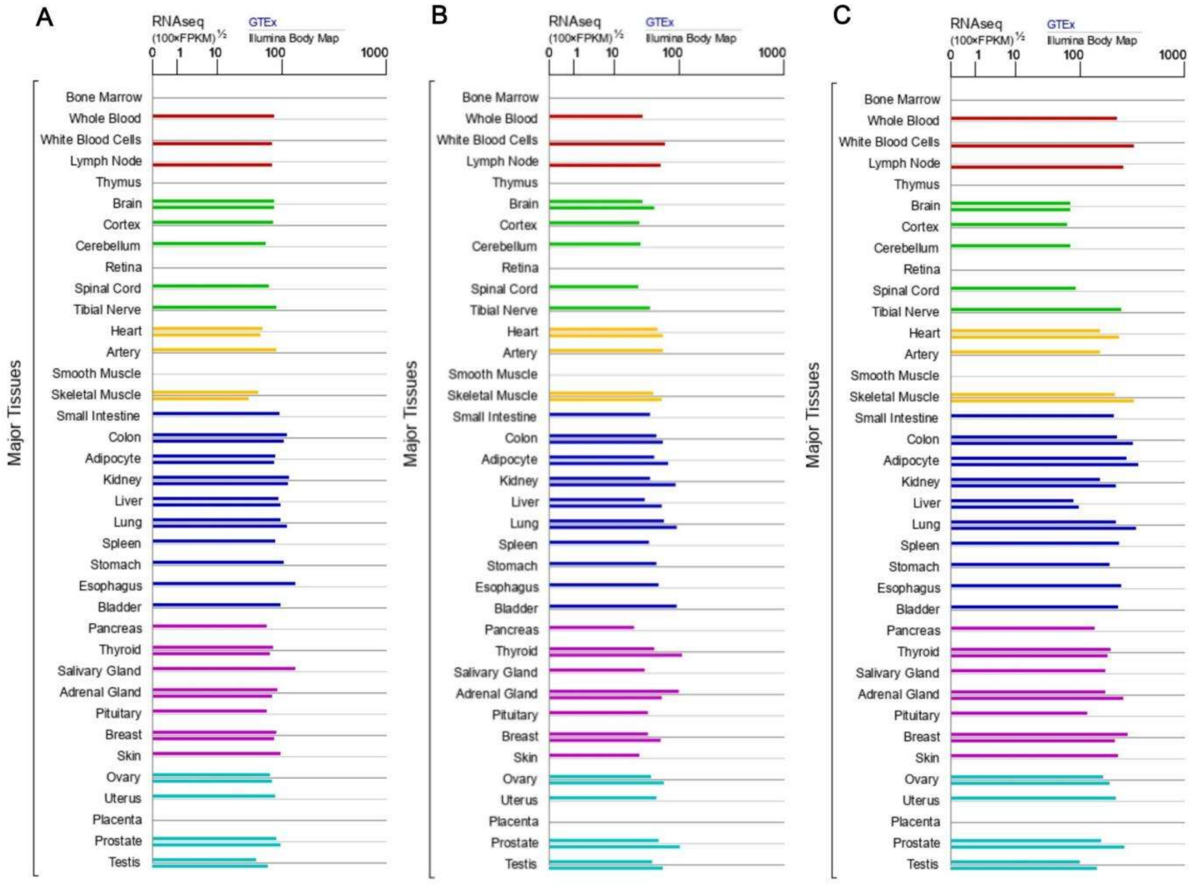

Supplementary Figure 1. The expression levels of the Trx system. The mRNA expression levels of TXN (A), TXNIP (B), and TXNRD1 (C) in various normal human tissues based on the GTEx database.

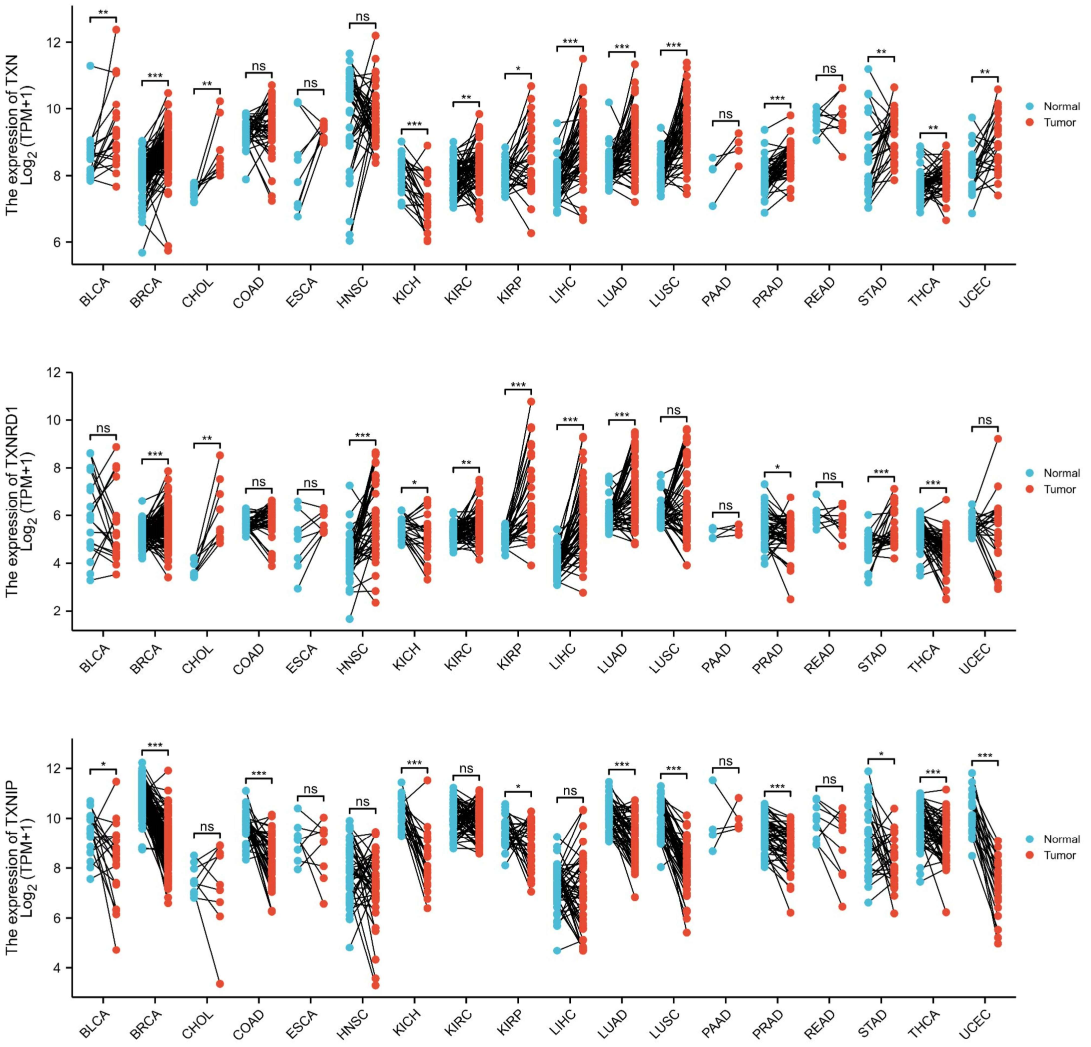

Supplementary Figure 2. Related to Figure 2. The expression of Trx system genes in pan-cancer. The mRNA expression levels of TXN, TXNIP, and TXNRD1 in tumor and adjacent normal tissues obtained from TCGA database.

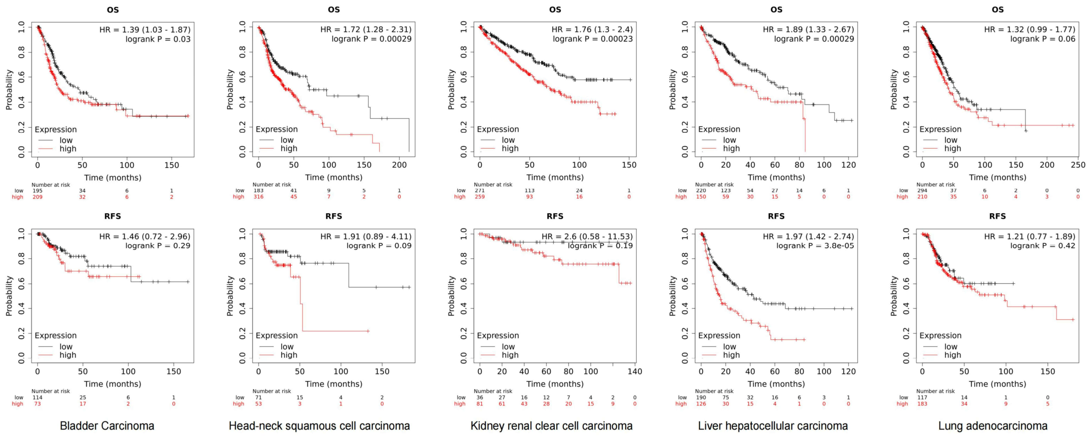

Supplementary Figure 3. Kaplan-Meier survival analysis of the selected genes in pan-cancer. Related to Figure 4. Survival analysis of genes in malignant tumors including the bladder, head and neck, kidney, liver, and lung cancer.

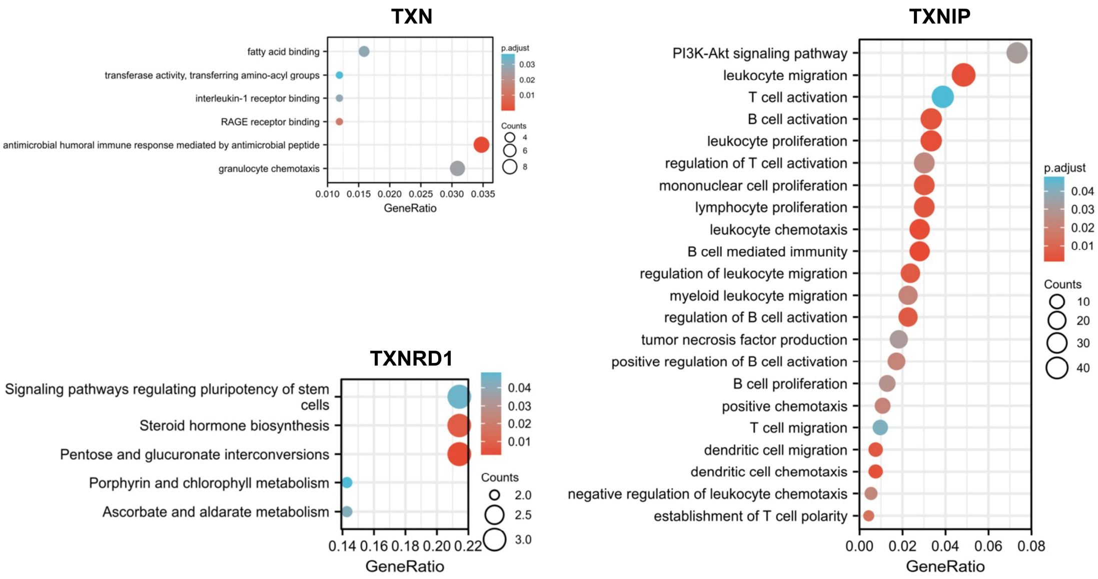

Supplementary Figure 4. Enrichment Analysis of the Trx system genes. Related to Figure 5. The enrichment of GO and KEGG terms by GSEA of selected genes in gastric cancer.

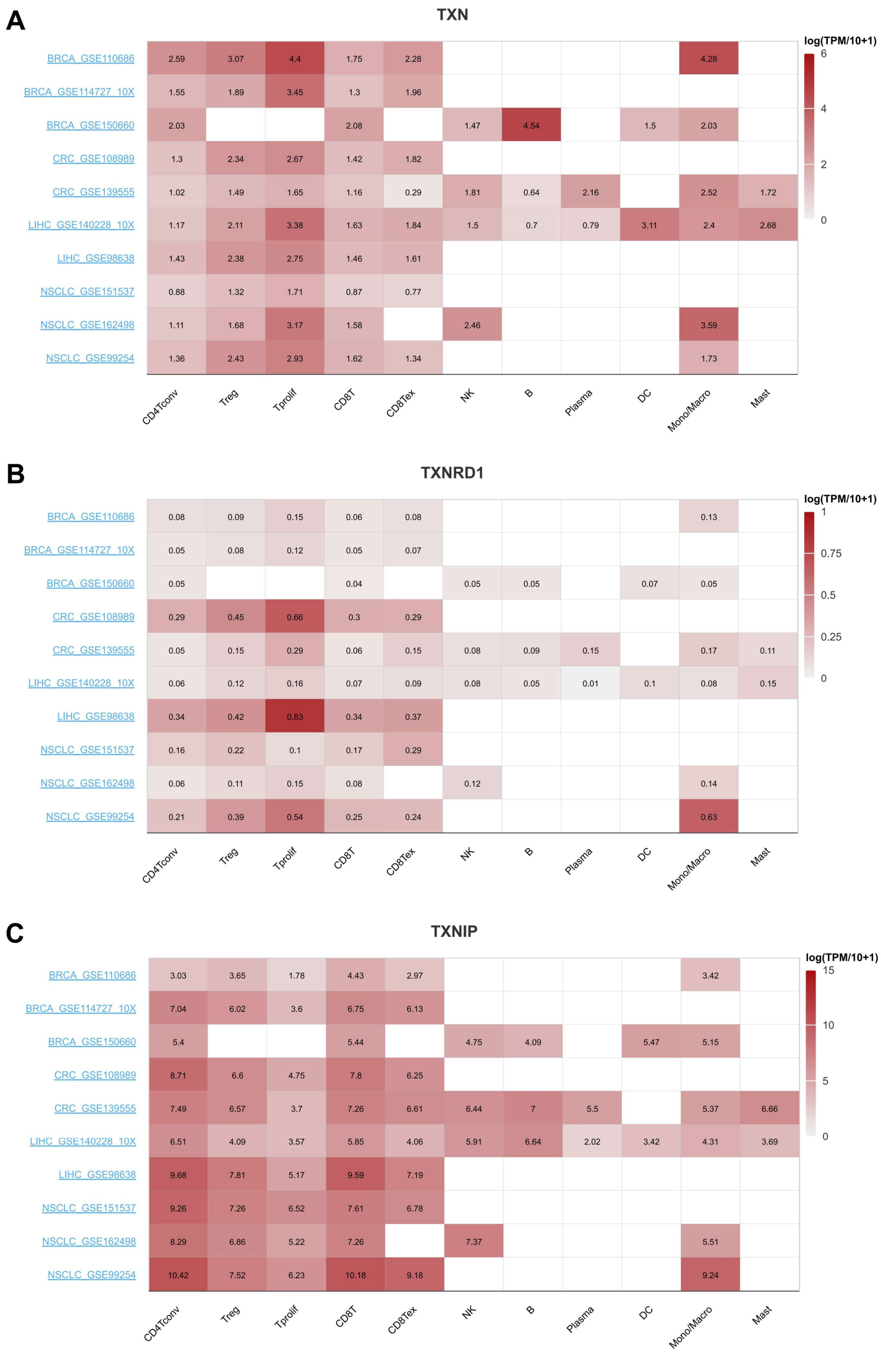

Supplementary Figure 5. Single-cell analysis of Trx system genes in immune cells. Related to Figure 6. Heatmaps showing the expression pattern of TXN, TXNRD1, TXNIP in immune cell at single-cell revolution.

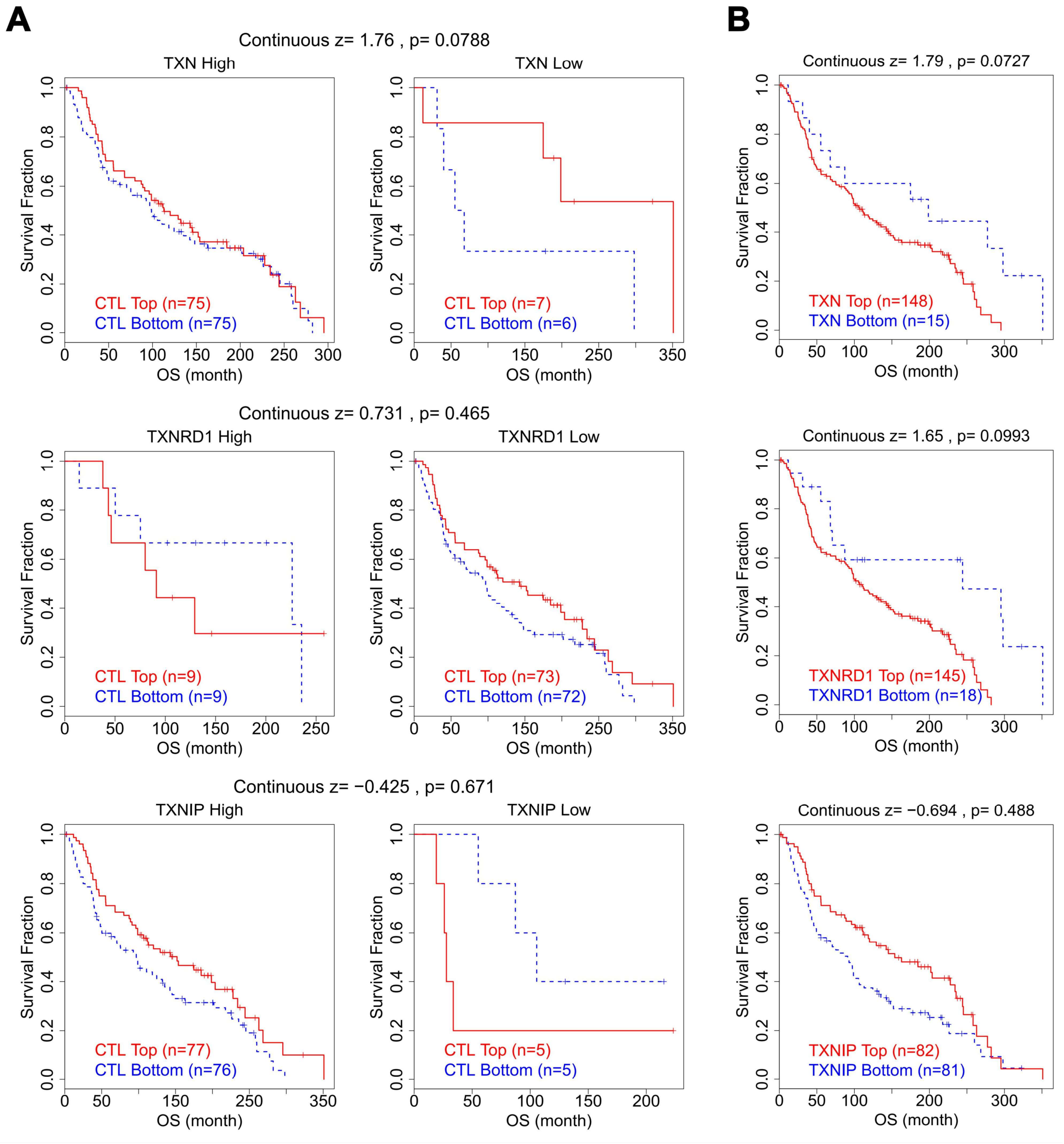

Supplementary Figure 6. The Correlation Analysis between Trx System and T Cell Dysfunction in BRCA. Related to Figure 7. (A) The survival curves illustrated the relationship between dysfunctional T cell phenotype and prognosis in different expression levels of Trx system in BRCA. (B) The survival curves illustrated the association between the expression of Trx system genes and prognosis in dysfunctional T cell phenotype in BRCA.

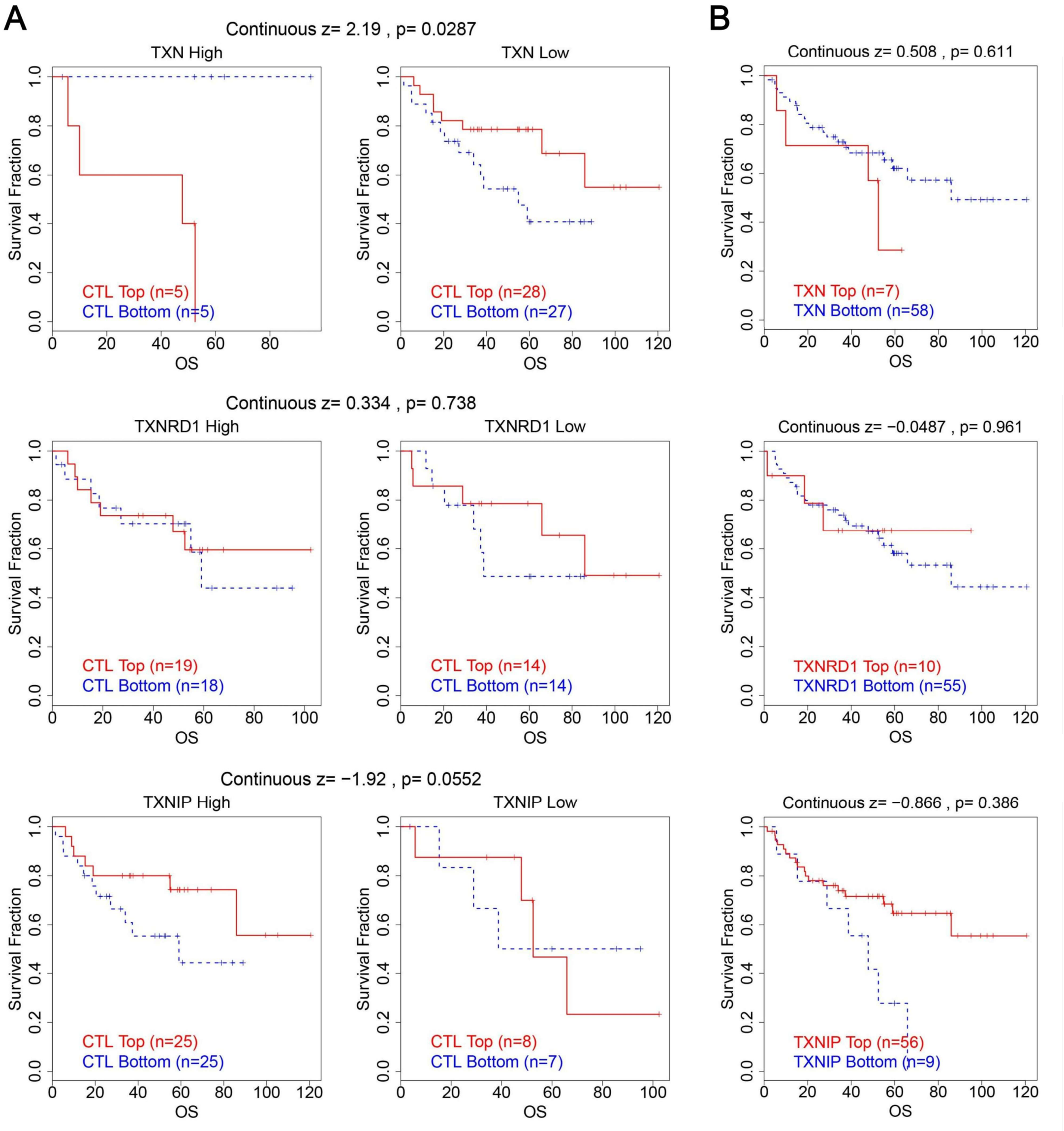

Supplementary Figure 7. The Correlation Analysis between Trx System and T Cell Dysfunction in COAD. Related to Figure 7. (A) The survival curves illustrated the relationship between dysfunctional T cell phenotype and prognosis in different expression levels of Trx system in COAD. (B) The survival curves illustrated the association between the expression of Trx system genes and prognosis in dysfunctional T cell phenotype in COAD.

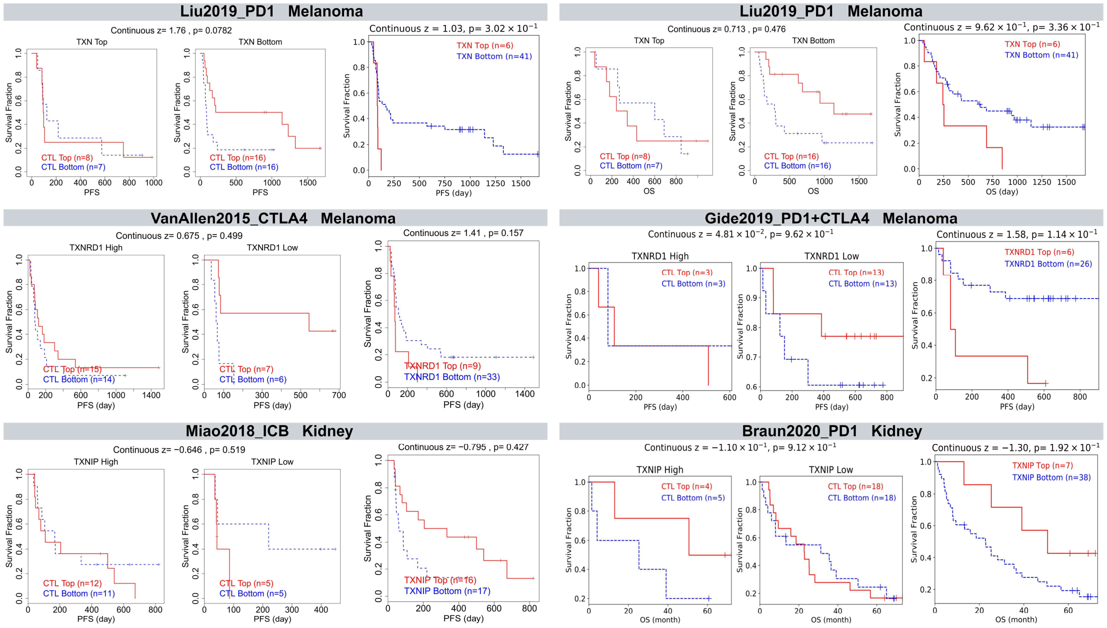

Supplementary Figure 8. Assessment of the association between Trx system and T cell dysfunction in ICB sub-cohorts. Related to Figure 8. Kaplan-Meier survival analysis illustrated the relationship between Trx system expression and T cell dysfunction and overall survival in other ICB sub-cohorts corresponding to sections in Figure 8C.
